# *trans*,*trans*,*trans*-Diaquabis(nicotinamide-κ*N*)bis­(2-nitro­benzoato-κ*O*)cadmium(II) dihydrate

**DOI:** 10.1107/S1600536809005479

**Published:** 2009-02-21

**Authors:** Kou-Lin Zhang, Bo Yang, Jian-Guo Lin, Seik Weng Ng

**Affiliations:** a College of Chemistry and Chemical Engineering, Yangzhou University, Yangzhou 225002, People’s Republic of China; b Department of Chemistry, University of Malaya, 50603, Kuala Lumpur, Malaysia

## Abstract

The cadmium atom in the title compound, [Cd(C_7_H_4_NO_4_)_2_(C_6_H_6_N_2_O)_2_(H_2_O)_2_]·2H_2_O, lies on a center of inversion in an all-*trans* octa­hedral environment. In the crystal, the complex inter­acts with the uncoordinated water mol­ecules through O—H⋯O and N—H⋯O hydrogen bonds, forming a layered network.

## Related literature

There are several examples of diaquadi(aryl­carboxyl­ato)di(nicotinamide)metal(II) compounds. For recent examples, see: Hökelek & Necefoğlu (2007*a* ▶,*b* ▶); Hökelek *et al.* (2007 ▶); Koksharova *et al.* (2006 ▶); Şahin *et al.* (2007*a* ▶,*b* ▶); Stachova *et al.* (2006 ▶); Çaylak *et al.* (2007 ▶).
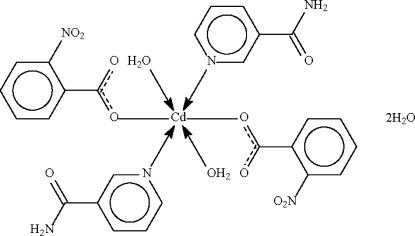


## Experimental

### 

#### Crystal data

[Cd(C_7_H_4_NO_4_)_2_(C_6_H_6_N_2_O)_2_(H_2_O)_2_]·2H_2_O
*M* *_r_* = 760.94
Monoclinic, 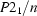

*a* = 7.9365 (8) Å
*b* = 19.589 (2) Å
*c* = 10.059 (1) Å
β = 103.178 (2)°
*V* = 1522.6 (3) Å^3^
*Z* = 2
Mo *K*α radiation
μ = 0.80 mm^−1^
*T* = 293 K
0.50 × 0.18 × 0.18 mm

#### Data collection

Bruker SMART area-detector diffractometer
Absorption correction: multi-scan (*SADABS*; Sheldrick, 1996 ▶) *T*_min_ = 0.619, *T*_max_ = 0.866
4427 measured reflections
2651 independent reflections
2396 reflections with *I* > 2σ(*I*)
*R*_int_ = 0.016

#### Refinement

*R*[*F* ^2^ > 2σ(*F* ^2^)] = 0.034
*wR*(*F* ^2^) = 0.093
*S* = 1.13
2651 reflections
246 parameters
9 restraints
H atoms treated by a mixture of independent and constrained refinement
Δρ_max_ = 0.42 e Å^−3^
Δρ_min_ = −1.04 e Å^−3^

### 

Data collection: *SMART* (Bruker, 2000 ▶); cell refinement: *SAINT* (Bruker, 2000 ▶); data reduction: *SAINT*; program(s) used to solve structure: *SHELXS97* (Sheldrick, 2008 ▶); program(s) used to refine structure: *SHELXL97* (Sheldrick, 2008 ▶); molecular graphics: *X-SEED* (Barbour, 2001 ▶); software used to prepare material for publication: *publCIF* (Westrip, 2009 ▶).

## Supplementary Material

Crystal structure: contains datablocks global, I. DOI: 10.1107/S1600536809005479/pv2136sup1.cif

Structure factors: contains datablocks I. DOI: 10.1107/S1600536809005479/pv2136Isup2.hkl

Additional supplementary materials:  crystallographic information; 3D view; checkCIF report

## Figures and Tables

**Table 1:** Hydrogen-bond geometry (Å, °)

*D*—H⋯*A*	*D*—H	H⋯*A*	*D*⋯*A*	*D*—H⋯*A*
O1w—H11⋯O2w	0.85 (4)	1.92 (4)	2.764 (4)	174 (4)
O1w—H12⋯O2^i^	0.85 (4)	1.95 (5)	2.718 (4)	150 (4)
O2w—H21⋯O5^i^	0.85 (3)	2.08 (3)	2.910 (4)	166 (4)
O2w—H22⋯O1^ii^	0.85 (3)	2.00 (1)	2.846 (3)	177 (5)
N3—H31⋯O2^iii^	0.85 (3)	2.22 (2)	3.038 (4)	165 (4)
N3—H32⋯O5^iv^	0.85 (3)	2.05 (3)	2.873 (4)	164 (4)

Symmetry codes: (i) 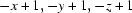; (ii) 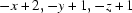; (iii) 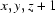; (iv) 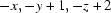.

**Fig. 1.**
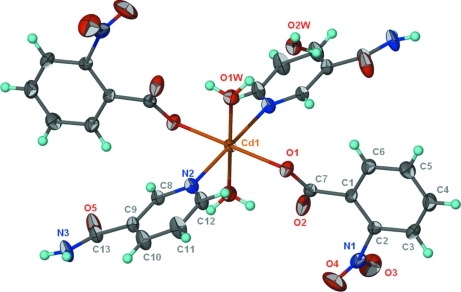
Thermal ellipsoid plot of Cd(H2O)2(C7H4NO4)2(C6H6N2O)2.2H2O; displacement ellipsoids are drawn at the 50% probabability level, and H atoms as spheres of arbitrary radii.

## supplementary crystallographic information

### Experimental

A water/methanol (1:1 *v*/*v*) solution (3 ml) of cadmium nitrate trihydrate (0.082 g, 0.3 mmol) was added to a water/methanol (1:1 *v*/*v*) solution (3 ml) of 2-nitrobenzoic acid (0.100 g, 0.6 mmol), sodium hydroxide (0.024 g, 0.6 mmol) and nicotinamide (0.073 g, 0.6 mmol). A white powder was obtained after several days; this was recrystallized from DMF/methanol (3:1 *v*/*v*) to give colorless crystals in 50% yield. CH&N elemental analysis. Calculated for C_26_H_28_CdN_6_O_14_: C 41.04 H 3.68 N 11.04%; found: C 40.08, H 3.87, N 10.93%.

### Refinement

Carbon-bound H atoms were placed in calculated positions and were allowed to ride on the parent atoms. N and O-bound H atoms were located in a difference Fourier map, and were refined with distance restraints N–H = O–H = 0.85±0.01 Å; for the water molecules, an additional H···H 1.39±0.01 Å restraint was used. Their temperature factors were freely refined.

The measurements are 100% at the 2θ limit of 50 °.

### Crystal data

**Table:**

[Cd(C_7_H_4_NO_4_)_2_(C_6_H_6_N_2_O)_2_(H_2_O)_2_]·2H_2_O	*F*(000) = 772
*M**_r_* = 760.94	*D*_x_ = 1.660 Mg m^−^^3^
Monoclinic, *P*2_1_/*n*	Mo *K*α radiation, λ = 0.71073 Å
Hall symbol: -P 2yn	Cell parameters from 3417 reflections
*a* = 7.9365 (8) Å	θ = 2.1–25.1°
*b* = 19.589 (2) Å	µ = 0.80 mm^−^^1^
*c* = 10.059 (1) Å	*T* = 293 K
β = 103.178 (2)°	Rod, colorless
*V* = 1522.6 (3) Å^3^	0.50 × 0.18 × 0.18 mm
*Z* = 2	

### Data collection

**Table:**

Bruker SMART area-detector diffractometer	2651 independent reflections
Radiation source: medium-focus sealed tube	2396 reflections with *I* > 2σ(*I*)
graphite	*R*_int_ = 0.016
φ and ω scans	θ_max_ = 25.1°, θ_min_ = 2.1°
Absorption correction: multi-scan (*SADABS*; Sheldrick, 1996)	*h* = −9→3
*T*_min_ = 0.619, *T*_max_ = 0.866	*k* = −20→23
4427 measured reflections	*l* = −11→11

### Refinement

**Table:**

Refinement on *F*^2^	Primary atom site location: structure-invariant direct methods
Least-squares matrix: full	Secondary atom site location: difference Fourier map
*R*[*F*^2^ > 2σ(*F*^2^)] = 0.034	Hydrogen site location: inferred from neighbouring sites
*wR*(*F*^2^) = 0.093	H atoms treated by a mixture of independent and constrained refinement
*S* = 1.13	*w* = 1/[σ^2^(*F*_o_^2^) + (0.0433*P*)^2^ + 2.3534*P*] where *P* = (*F*_o_^2^ + 2*F*_c_^2^)/3
2651 reflections	(Δ/σ)_max_ = 0.001
246 parameters	Δρ_max_ = 0.42 e Å^−^^3^
9 restraints	Δρ_min_ = −1.04 e Å^−^^3^

### Fractional atomic coordinates and isotropic or equivalent isotropic displacement parameters (Å^2^)

**Table:**

	*x*	*y*	*z*	*U*_iso_*/*U*_eq_	
Cd1	0.5000	0.5000	0.5000	0.02349 (13)	
O1	0.6570 (3)	0.40982 (12)	0.4385 (2)	0.0312 (5)	
O2	0.4256 (3)	0.36571 (14)	0.2970 (3)	0.0428 (7)	
O3	0.3595 (4)	0.23243 (19)	0.1217 (4)	0.0693 (10)	
O4	0.4552 (5)	0.2124 (2)	0.3355 (4)	0.0835 (12)	
O5	0.0789 (4)	0.48477 (17)	0.8538 (3)	0.0505 (8)	
O1W	0.7685 (3)	0.55083 (13)	0.5819 (3)	0.0362 (6)	
H11	0.842 (5)	0.561 (2)	0.536 (4)	0.055 (14)*	
H12	0.737 (6)	0.5868 (14)	0.616 (5)	0.082 (19)*	
O2W	0.9923 (3)	0.57871 (14)	0.4149 (3)	0.0375 (6)	
H21	0.987 (5)	0.556 (2)	0.342 (3)	0.079 (18)*	
H22	1.097 (2)	0.581 (2)	0.460 (3)	0.050 (13)*	
N1	0.4713 (4)	0.23414 (16)	0.2260 (4)	0.0438 (8)	
N2	0.5129 (3)	0.44979 (15)	0.7119 (3)	0.0291 (6)	
N3	0.1940 (4)	0.44256 (19)	1.0627 (3)	0.0405 (8)	
H31	0.274 (4)	0.424 (2)	1.122 (3)	0.055 (13)*	
H32	0.114 (4)	0.457 (2)	1.098 (3)	0.049 (12)*	
C1	0.6994 (4)	0.32348 (16)	0.2832 (3)	0.0237 (6)	
C2	0.6421 (4)	0.26190 (17)	0.2198 (3)	0.0282 (7)	
C3	0.7386 (5)	0.22316 (19)	0.1503 (4)	0.0386 (9)	
H3	0.6942	0.1829	0.1070	0.047 (12)*	
C4	0.9033 (5)	0.2454 (2)	0.1459 (4)	0.0407 (9)	
H4	0.9714	0.2196	0.1008	0.047 (12)*	
C5	0.9655 (5)	0.3057 (2)	0.2087 (4)	0.0430 (9)	
H5	1.0761	0.3205	0.2063	0.057 (13)*	
C6	0.8643 (4)	0.34455 (19)	0.2755 (4)	0.0337 (8)	
H6	0.9075	0.3855	0.3161	0.046 (12)*	
C7	0.5842 (4)	0.36885 (16)	0.3458 (3)	0.0262 (7)	
C8	0.3792 (4)	0.46351 (17)	0.7674 (3)	0.0256 (7)	
H8	0.2975	0.4951	0.7238	0.044 (12)*	
C9	0.3553 (4)	0.43356 (17)	0.8857 (3)	0.0273 (7)	
C10	0.4767 (5)	0.3864 (2)	0.9499 (4)	0.0399 (9)	
H10	0.4645	0.3648	1.0295	0.044 (11)*	
C11	0.6165 (5)	0.3721 (2)	0.8941 (4)	0.0461 (10)	
H11A	0.7004	0.3410	0.9362	0.055 (13)*	
C12	0.6301 (5)	0.40434 (19)	0.7751 (4)	0.0352 (8)	
H12A	0.7239	0.3942	0.7375	0.052 (12)*	
C13	0.1972 (4)	0.45472 (18)	0.9337 (3)	0.0312 (7)	

### Atomic displacement parameters (Å^2^)

**Table:**

	*U*^11^	*U*^22^	*U*^33^	*U*^12^	*U*^13^	*U*^23^
Cd1	0.02157 (19)	0.0277 (2)	0.0224 (2)	−0.00033 (12)	0.00743 (13)	0.00046 (12)
O1	0.0292 (12)	0.0324 (13)	0.0303 (12)	0.0020 (10)	0.0034 (10)	−0.0075 (10)
O2	0.0268 (13)	0.0550 (17)	0.0443 (15)	0.0049 (12)	0.0033 (11)	−0.0207 (13)
O3	0.0398 (17)	0.078 (2)	0.085 (3)	−0.0141 (16)	0.0042 (17)	−0.027 (2)
O4	0.087 (3)	0.098 (3)	0.078 (3)	−0.041 (2)	0.044 (2)	0.003 (2)
O5	0.0388 (16)	0.084 (2)	0.0318 (15)	0.0297 (15)	0.0135 (12)	0.0122 (14)
O1W	0.0273 (13)	0.0406 (14)	0.0416 (15)	−0.0066 (11)	0.0094 (11)	−0.0085 (12)
O2W	0.0276 (13)	0.0452 (15)	0.0390 (15)	0.0019 (11)	0.0061 (11)	−0.0039 (12)
N1	0.0417 (19)	0.0363 (18)	0.057 (2)	−0.0114 (14)	0.0190 (17)	−0.0155 (16)
N2	0.0251 (14)	0.0348 (15)	0.0274 (15)	0.0037 (12)	0.0059 (11)	0.0027 (12)
N3	0.0366 (17)	0.066 (2)	0.0223 (15)	0.0158 (16)	0.0128 (13)	0.0093 (15)
C1	0.0281 (16)	0.0240 (16)	0.0173 (14)	0.0011 (13)	0.0015 (12)	−0.0017 (12)
C2	0.0282 (17)	0.0258 (17)	0.0313 (17)	−0.0024 (13)	0.0083 (14)	−0.0016 (14)
C3	0.051 (2)	0.0263 (18)	0.041 (2)	−0.0017 (16)	0.0154 (17)	−0.0088 (16)
C4	0.042 (2)	0.040 (2)	0.045 (2)	0.0102 (17)	0.0185 (17)	−0.0024 (17)
C5	0.0305 (19)	0.047 (2)	0.056 (3)	−0.0013 (17)	0.0203 (18)	−0.0033 (19)
C6	0.0303 (18)	0.0349 (19)	0.0359 (19)	−0.0044 (15)	0.0075 (15)	−0.0083 (15)
C7	0.0254 (16)	0.0243 (16)	0.0297 (17)	0.0019 (13)	0.0078 (13)	0.0013 (13)
C8	0.0246 (16)	0.0309 (18)	0.0201 (15)	0.0033 (13)	0.0025 (12)	0.0029 (13)
C9	0.0282 (17)	0.0336 (18)	0.0199 (15)	0.0028 (14)	0.0051 (13)	−0.0012 (13)
C10	0.042 (2)	0.053 (2)	0.0267 (18)	0.0173 (18)	0.0117 (15)	0.0150 (17)
C11	0.041 (2)	0.062 (3)	0.035 (2)	0.027 (2)	0.0095 (17)	0.0190 (19)
C12	0.0303 (18)	0.046 (2)	0.0317 (19)	0.0088 (16)	0.0117 (15)	0.0022 (16)
C13	0.0304 (18)	0.0375 (19)	0.0274 (17)	0.0051 (15)	0.0098 (14)	0.0027 (14)

### Geometric parameters (Å, °)

**Table:**

Cd1—O1^i^	2.325 (2)	C1—C2	1.391 (4)
Cd1—O1	2.325 (2)	C1—C6	1.391 (5)
Cd1—O1W^i^	2.326 (2)	C1—C7	1.512 (4)
Cd1—O1W	2.326 (2)	C2—C3	1.377 (5)
Cd1—N2	2.329 (3)	C3—C4	1.388 (5)
Cd1—N2^i^	2.329 (3)	C3—H3	0.9300
O1—C7	1.266 (4)	C4—C5	1.378 (6)
O2—C7	1.244 (4)	C4—H4	0.9300
O3—N1	1.211 (5)	C5—C6	1.386 (5)
O4—N1	1.214 (5)	C5—H5	0.9300
O5—C13	1.237 (4)	C6—H6	0.9300
O1W—H11	0.85 (4)	C8—C9	1.378 (5)
O1W—H12	0.85 (4)	C8—H8	0.9300
O2W—H21	0.85 (3)	C9—C10	1.383 (5)
O2W—H22	0.85 (3)	C9—C13	1.502 (5)
N1—C2	1.476 (4)	C10—C11	1.382 (5)
N2—C8	1.334 (4)	C10—H10	0.9300
N2—C12	1.339 (4)	C11—C12	1.379 (5)
N3—C13	1.325 (4)	C11—H11A	0.9300
N3—H31	0.84 (3)	C12—H12A	0.9300
N3—H32	0.85 (3)		
			
O1^i^—Cd1—O1	180.000 (1)	C1—C2—N1	120.5 (3)
O1^i^—Cd1—O1W^i^	85.20 (9)	C2—C3—C4	118.7 (3)
O1—Cd1—O1W^i^	94.80 (9)	C2—C3—H3	120.7
O1^i^—Cd1—O1W	94.80 (9)	C4—C3—H3	120.7
O1—Cd1—O1W	85.20 (9)	C5—C4—C3	119.8 (3)
O1W^i^—Cd1—O1W	180.00 (12)	C5—C4—H4	120.1
O1^i^—Cd1—N2	89.52 (9)	C3—C4—H4	120.1
O1—Cd1—N2	90.48 (9)	C4—C5—C6	120.4 (3)
O1W^i^—Cd1—N2	89.36 (10)	C4—C5—H5	119.8
O1W—Cd1—N2	90.64 (10)	C6—C5—H5	119.8
O1^i^—Cd1—N2^i^	90.48 (9)	C5—C6—C1	121.4 (3)
O1—Cd1—N2^i^	89.52 (9)	C5—C6—H6	119.3
O1W^i^—Cd1—N2^i^	90.64 (10)	C1—C6—H6	119.3
O1W—Cd1—N2^i^	89.36 (10)	O2—C7—O1	125.0 (3)
N2—Cd1—N2^i^	180.0	O2—C7—C1	117.4 (3)
C7—O1—Cd1	119.7 (2)	O1—C7—C1	117.5 (3)
Cd1—O1W—H11	126 (3)	N2—C8—C9	123.7 (3)
Cd1—O1W—H12	100 (3)	N2—C8—H8	118.2
H11—O1W—H12	109 (4)	C9—C8—H8	118.2
H21—O2W—H22	109.4 (17)	C8—C9—C10	118.0 (3)
O3—N1—O4	124.7 (4)	C8—C9—C13	116.7 (3)
O3—N1—C2	118.2 (4)	C10—C9—C13	125.3 (3)
O4—N1—C2	117.1 (4)	C11—C10—C9	118.9 (3)
C8—N2—C12	117.9 (3)	C11—C10—H10	120.5
C8—N2—Cd1	115.1 (2)	C9—C10—H10	120.5
C12—N2—Cd1	126.6 (2)	C12—C11—C10	119.3 (3)
C13—N3—H31	126 (3)	C12—C11—H11A	120.4
C13—N3—H32	122 (2)	C10—C11—H11A	120.4
H31—N3—H32	111.4 (18)	N2—C12—C11	122.2 (3)
C2—C1—C6	116.4 (3)	N2—C12—H12A	118.9
C2—C1—C7	122.4 (3)	C11—C12—H12A	118.9
C6—C1—C7	121.0 (3)	O5—C13—N3	122.8 (3)
C3—C2—C1	123.3 (3)	O5—C13—C9	119.2 (3)
C3—C2—N1	116.2 (3)	N3—C13—C9	118.0 (3)
			
O1W^i^—Cd1—O1—C7	22.2 (2)	C4—C5—C6—C1	1.0 (6)
O1W—Cd1—O1—C7	−157.8 (2)	C2—C1—C6—C5	−0.3 (5)
N2—Cd1—O1—C7	111.6 (2)	C7—C1—C6—C5	−174.8 (3)
N2^i^—Cd1—O1—C7	−68.4 (2)	Cd1—O1—C7—O2	−11.8 (5)
O1^i^—Cd1—N2—C8	29.7 (2)	Cd1—O1—C7—C1	163.9 (2)
O1—Cd1—N2—C8	−150.3 (2)	C2—C1—C7—O2	−27.5 (5)
O1W^i^—Cd1—N2—C8	−55.5 (2)	C6—C1—C7—O2	146.7 (3)
O1W—Cd1—N2—C8	124.5 (2)	C2—C1—C7—O1	156.4 (3)
O1^i^—Cd1—N2—C12	−157.2 (3)	C6—C1—C7—O1	−29.4 (4)
O1—Cd1—N2—C12	22.8 (3)	C12—N2—C8—C9	0.0 (5)
O1W^i^—Cd1—N2—C12	117.6 (3)	Cd1—N2—C8—C9	173.7 (3)
O1W—Cd1—N2—C12	−62.4 (3)	N2—C8—C9—C10	−0.3 (5)
C6—C1—C2—C3	−1.2 (5)	N2—C8—C9—C13	−179.8 (3)
C7—C1—C2—C3	173.2 (3)	C8—C9—C10—C11	0.7 (6)
C6—C1—C2—N1	178.0 (3)	C13—C9—C10—C11	−179.9 (4)
C7—C1—C2—N1	−7.5 (5)	C9—C10—C11—C12	−0.8 (7)
O3—N1—C2—C3	−69.1 (5)	C8—N2—C12—C11	−0.1 (6)
O4—N1—C2—C3	108.4 (4)	Cd1—N2—C12—C11	−173.0 (3)
O3—N1—C2—C1	111.6 (4)	C10—C11—C12—N2	0.6 (7)
O4—N1—C2—C1	−70.9 (5)	C8—C9—C13—O5	15.8 (5)
C1—C2—C3—C4	1.9 (6)	C10—C9—C13—O5	−163.7 (4)
N1—C2—C3—C4	−177.4 (3)	C8—C9—C13—N3	−161.5 (3)
C2—C3—C4—C5	−1.1 (6)	C10—C9—C13—N3	19.0 (6)
C3—C4—C5—C6	−0.3 (6)		

Symmetry codes: (i) −*x*+1, −*y*+1, −*z*+1.

### Hydrogen-bond geometry (Å, °)

**Table:**

*D*—H···*A*	*D*—H	H···*A*	*D*···*A*	*D*—H···*A*
O1w—H11···O2w	0.85 (4)	1.92 (4)	2.764 (4)	174 (4)
O1w—H12···O2^i^	0.85 (4)	1.95 (5)	2.718 (4)	150 (4)
O2w—H21···O5^i^	0.85 (3)	2.08 (3)	2.910 (4)	166 (4)
O2w—H22···O1^ii^	0.85 (3)	2.00 (1)	2.846 (3)	177 (5)
N3—H31···O2^iii^	0.85 (3)	2.22 (2)	3.038 (4)	165 (4)
N3—H32···O5^iv^	0.85 (3)	2.05 (3)	2.873 (4)	164 (4)

Symmetry codes: (i) −*x*+1, −*y*+1, −*z*+1; (ii) −*x*+2, −*y*+1, −*z*+1; (iii) *x*, *y*, *z*+1; (iv) −*x*, −*y*+1, −*z*+2.
